# Efficacy of L-methylfolate and methylcobalamin in treating resistant hypertension associated with elevated serum homocysteine in hemodialysis patients

**DOI:** 10.1186/s12882-025-04726-8

**Published:** 2026-01-24

**Authors:** Mohamed Sherif Salem, Noha Alaa Hamdy, Hesham Abdallah Elghoneimy, Hanan MS El Gowelli

**Affiliations:** 1 Clinical Pharmacy & Pharmacy Practice Department, Faculty of Pharmacy, Alexandria University, Alexandria, Egypt; 2 Andalusia Shalalat Hospital, Alexandria, Egypt; 3 Nephrology Unit, Internal Medicine Department, Faculty of Medicine, Alexandria University, Alexandria, Egypt; 4 Pharmacology and Toxicology Department, Faculty of Pharmacy, Alexandria University, Alexandria, Egypt

**Keywords:** Hypertension, End-stage renal disease, Hyperhomocysteinemia, Resistant hypertension, L-methylfolate, Methylcobalamin

## Abstract

**Background:**

End-stage renal disease (ESRD) patients receiving hemodialysis are experiencing a considerable increase in the burden of cardiovascular diseases (CVDs). In this patient population, hypertension is a prevalent modifiable cardiovascular risk factor that is associated with poor prognosis. Resistant hypertension in dialysis patients is challenging to manage since some individuals do not respond to antihypertensive medications or volume control. Hyperhomocysteinemia is common among ESRD patients. “H-type hypertension” or hyperhomocysteinemia-associated hypertension refers to resistant hypertension with elevated cardiovascular risk. The current study examined the efficacy of methylfolate and methylcobalamin supplementation in reducing serum homocysteine levels and improving blood pressure (BP) control in ESRD patients with resistant hypertension on regular hemodialysis.

**Methods:**

Throughout the study, 51 ESRD patients with resistant hypertension were randomly allocated to receive either daily doses of L-methylfolate 800 mcg and methylcobalamin 1000 mcg capsule (intervention group) or no medication (control group). Serum homocysteine levels were measured twice: at baseline and three months later. In addition, average pre- and post-dialysis blood pressure readings were obtained at baseline, one month, two months, and three months.

**Results:**

After three months, mean serum homocysteine levels were significantly lower than at the commencement of therapy (*p* = 0.035), nonetheless, control patients showed no significant difference. Between-group analysis found a statistically significant difference in the change in homocysteine levels among the two groups (*p* = 0.006). Furthermore, the treatment group had statistically significant lower pre- and post-dialysis blood pressure readings.

**Conclusions:**

A three-month supplementation with a combination of 800 mcg methylfolate and 1000 mcg methylcobalamin showed promise in lowering blood pressure and serum homocysteine levels in ESRD patients with resistant hypertension. These findings require additional exploration in larger studies.

**Trial registration:**

ClinicalTrials.gov Identifier NCT05807711 registered on 20,230,329.

## Introduction

Patients with end-stage renal disease (ESRD) have a high incidence of concomitant hypertension. The main reason for the variation in the prevalence of hypertension across studies is the fact that different researchers may define hypertension distinctly and utilize different methodologies for measuring blood pressure either before, after hemodialysis or by ambulatory blood pressure recordings. Of note, about 50 to 60% of patients on hemodialysis are hypertensive, with some reports indicating the percentage to be 85% [1–3]. 

In dialysis patients, volume expansion contributes mostly to hypertension through increased accumulation of extracellular fluid, which causes an increase in BP. This phenomenon leads to high BP with two main mechanisms: increased cardiac output and systemic vascular resistance [4–6]. Furthermore, there is supportive evidence that the combined effects of increased sympathetic nervous system activity, renin-angiotensin stimulation, and arteriosclerosis can become an incentive for the development of arterial hypertension in hemodialysis patients [7, 8]. Other possible etiologies for resistant hypertension in these patients are the use of erythropoiesis-stimulating agents (ESA) and over-the-counter medications such as nasal decongestants and non-steroidal anti-inflammatory drugs [9]. 

Interventions for resistant hypertension among patients on dialysis focus, first, on targeting dry weight adjustment to achieve euvolemia and, secondly, on antihypertensive agents [5, 10, 11]. Nevertheless, a subset of the patients demonstrates an inadequate response to either volume management or standard antihypertensive agents. One main reason that contributes to this ongoing clinical challenge is the chronic nonadherence to prescribed antihypertensive medications [12]. 

Homocysteine is a non-proteinogenic amino acid generated by methionine catabolism via strictly regulated metabolic pathways governed by two key mechanisms: trans-methylation and trans-sulfuration. These mechanisms rely substantially on important micronutrients such as folate and vitamin B_12_ for appropriate functioning/metabolism [13, 14]. Normal homocysteine concentrations typically range between 5 and 15 micromol/L. Hyperhomocysteinemia is classified into three categories: moderate (15–30 micromol/L), intermediate (30–100 micromol/L), and severe (> 100 micromol/L) [15]. The elevated levels of homocysteine are mostly due to impaired renal function [16]. However, the increase in homocysteine levels in this context may not solely stem from compromised glomerular filtration efficiency. Alternatively, another hypothesis proposes that a variation in renal homocysteine metabolism could be a distinguishing feature in this population. Despite unbound homocysteine having a low molecular weight and being able to overcome the filtration barrier, roughly 90% of homocysteine in circulation is bound to proteins and cannot be removed efficiently by the kidneys [17]. 

H-type hypertension is hypertension with elevated plasma homocysteine concentrations greater than or equal to 10 µmol/L [18]. There is some evidence that hyperhomocysteinemia could be related to hypertension; however, the pathways linking the two conditions remain incompletely understood. Hyperhomocysteinemia is associated with endothelial dysfunction, oxidative stress, inflammation, and diminished nitric oxide bioavailability, which all play a direct role in the development of hypertension. In addition, hyperhomocysteinemia may also contribute to the vascular changes, including remodeling, stiffness, and initiation of atherosclerosis processes, and therefore worsening hypertension and its cardiovascular-related complications [19, 20]. 

In recent years, there has been growing interest in methylfolate as a potential alternative to folic acid for reducing serum homocysteine levels [21]. 

The present study aims to explore the potential effect of methylfolate and methylcobalamin supplementation in reducing serum homocysteine levels and enhancing blood pressure control among ESRD patients suffering from resistant hypertension and undergoing maintenance hemodialysis.

## Methodology

This study complied with the ethical guidelines outlined in the Declaration of Helsinki (1989) and received approval from the Ethics Committee on Human Research at the Faculty of Medicine, Alexandria University (Protocol ID: 0107591). At the beginning of the study, all participants provided written informed consent. Participants were informed about the research, including its potential risks and benefits, the voluntary nature of their participation, and their right to withdraw at any point.

### Study design & settings

This study was conducted as a single-center, open-label, randomized controlled trial at the Alexandria University Hemodialysis Unit between April and December 2023.

### Eligibility criteria

A total of 186 patients were screened for eligibility. The study included patients with ESRD who were hypertensive and exhibited resistant hypertension, defined as having an average pre-dialysis blood pressure exceeding 140/90 mm Hg and/or an average post-dialysis blood pressure above 130/80 mm Hg despite being on at least three antihypertensive medications based on evaluation of four mid-week sessions’ blood pressure values of a single month.

The standard definition of resistant hypertension in ambulatory patients usually includes a diuretic as one of the three antihypertensive classes. However, in maintenance hemodialysis populations, most patients are oliguric or anuric, and diuretics are often ineffective or not used. Therefore, we intentionally used a pragmatic dialysis-specific definition that does not require diuretic therapy. This approach aligns with previous dialysis hypertension literature that adjusts resistant-hypertension criteria for renal-replacement therapy populations [22, 23]. 

Exclusion criteria included patients older than 75 years of age, excessive use of alcohol or tobacco, severe hepatic impairment, non-dialysis dependent chronic kidney disease, pregnant women, known allergy to the study drug ingredients, and use of medications known to elevate serum homocysteine levels. A sample size of 50 patients was determined to be sufficient for this study, with equal allocation to the treatment group (N1 = 25) and control group (N2 = 25). This calculation was performed using G*Power 3.0.10 software, based on the following parameters: Alpha error (α): 0.05, indicating a 5% probability of falsely rejecting the null hypothesis. Power (1-β): 0.8, ensuring an 80% probability of correctly detecting a true effect, if it exists. Effect size: 0.72, representing a medium-to-large effect, based on relevant literature [24, 25] and Cohen’s d formula.

### Randomization

**Fig. 1:**
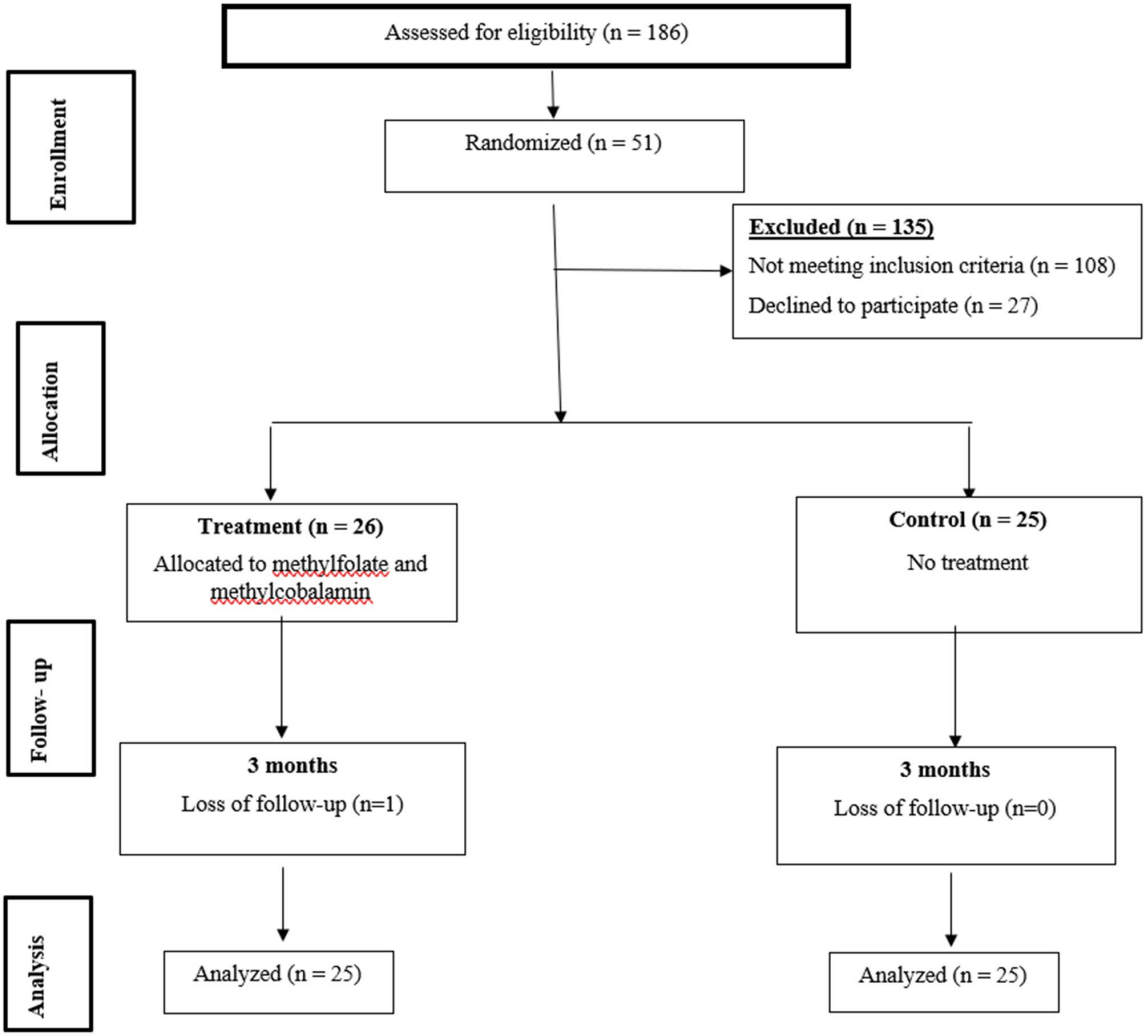
Flow chart of the study

Patients who were deemed eligible were randomized into two arms using a simple computer-generated program to take one tablet daily containing L-methylfolate and methylcobalamin (intervention group) or no treatment (control group). The flow chart of the studied patients is shown in Fig. 1.

### Baseline evaluation

Patients were evaluated for baseline characteristics, comorbidities, and medication history through medical record review and patients’ interviews. Demographic data, including age, weight, height, smoking history, medical history, and current drug list (including all antihypertensive medications), vascular access for dialysis, duration on dialysis, and etiology of ESRD were recorded. Baseline evaluation included blood pressure and laboratory measurements (homocysteine level, serum calcium, phosphorus, parathyroid hormone (PTH), and complete blood count (CBC)) were also retrieved. Anthropometric measurements were also calculated.

### Study outcomes

The main outcomes of this study were to assess both average pre-dialysis, post-dialysis blood pressure, and serum homocysteine at baseline and three months after drug intake in the treatment arm compared to the control group, who were receiving the standard of care.

### Blood pressure measurement protocol

In particular, a standardized protocol for blood pressure measurements was communicated to the attending nurses. The blood pressure readings were averaged based on four mid-week pre-dialysis and post-dialysis measurements taken using a calibrated sphygmomanometer. Patients were seated comfortably with their feet flat on the floor, backs supported, and arms positioned at heart level. The cuff was placed on the arm, aligning with the midpoint of the sternum. Patients were instructed to rest for at least five minutes without consuming caffeine and without exercise or smoking for 30 min before the measurement to minimize the impact of confounding factors. The averages of four mid-week pre-dialysis and post-dialysis blood pressure readings were measured for each participant at baseline, then after one, two, and three months.

Blood samples were collected at baseline and at three months in gel and clot tubes (VACUTEST^®^) and were immediately transferred on ice to a laboratory for analysis. Serum homocysteine concentrations were quantified within thirty minutes of collection using the Roche^®^ Homocysteine fully automated enzymatic assay. Antihypertensive medication regimens were maintained unless deemed necessary for clinical reasons to prevent severe hypertension or hypotension.

### Intervention arm

At the study enrollment, all participants were asked to stop taking any over-the-counter supplements containing folic acid or vitamin B12 for one month, except for the dialysis unit’s routine practice, which includes administration of a vitamin B-complex after dialysis composed of vitamin B3, B5, B6, B2, B1, and Biotin.

The medication used in the study, known as Methyl Folate^®^, comes in a hard gelatin capsule that contains 800 mcg of methylfolate and 1000 mcg of methylcobalamin.

After randomization, participants of the treatment group were asked to take one capsule of the study drug (methylfolate) every day for three months. To ensure compliance with the treatment, two strategies were employed; providing a month’s worth of supply at each follow-up visit and asking participants to return empty medication bottles during follow-up. The control group did not receive that treatment or any comparator.

For all of the patients included in the study, ultrafiltration goals were frequently adjusted, and every effort has been made to achieve the dry weight of the patients, including regular clinical examination, inferior vena cava assessment, and bioimpedance analysis. Antihypertensive medications were maintained whenever clinically safe; changes were permitted only for clinical indications (symptomatic hypotension or uncontrolled hypertension). Erythropoietin-stimulating agent (ESA) dosing followed routine nephrology care. ESA dose changes were allowed as per clinical indications. Every patient in the studied cohort received a thrice-weekly 4-hour session of hemodialysis using a Fresenius FX 80 high-flux hemodialyser for the first and the last sessions of the week and a Fresenius FX 10 low-flux hemodialyser for the mid-week session.

### Statistical analysis

Data were fed to the computer and analyzed using IBM SPSS Statistics software version 20.0 (Armonk, NY: IBM Corp.). Categorical data were represented as numbers and percentages. Chi-square tests were applied to compare between two groups. Alternatively, Fisher’s Exact test with a Monte Carlo correction was used when more than 20% of the cells had expected counts less than 5. The Shapiro-Wilk test was used to determine normality in continuous data. For normally distributed variables, quantitative data were reported as the range (minimum and maximum), mean, standard deviation, and median. We utilized Student’s t-test to compare between two independent groups, ANOVA with repeated measures to compare between the study periods, and a post-hoc test (modified Bonferroni) for pairwise comparisons. Paired t-tests were employed to compare baseline and follow-up data (3 months later). The Mann-Whitney U test was used to compare two groups of abnormally distributed quantitative variables, whereas the Wilcoxon signed-rank test was employed to compare baseline and follow-up data (after three months). All statistical tests have a significance threshold of 0.05. Finally, linear regression analysis revealed significant independent variables impacting homocysteine and blood pressure reduction.

## Results

Patients attending the Alexandria University Dialysis Unit between April and December 2023 were screened for eligibility to be enrolled in the study. One hundred and eight patients did not meet the inclusion criteria, and 27 patients declined to participate in the study. Fifty-one patients were randomized into two arms. The treatment group included 26 patients who received methylfolate and methylcobalamin in addition to their standard of care, while the other 25 patients were assigned to the control group, which received the standard of care only.

One patient in the intervention arm died from an acute myocardial infarction and was not included in the final analysis. Figure 1.

There were no statistically significant differences in the baseline characteristics, including gender, age, smoking status, anthropometric measures, vascular access, concomitant drugs, and etiology of ESRD between the two groups. The duration on dialysis was found to be significantly longer in the treatment group (*p* = 0.024) (Table 1).

**Table 1:** Comparison between baseline characteristics of the treatment and control groups

	Control (*n* = 25)	Treatment (*n* = 25)	Total (*n* = 50)	Test of Sig.	*P*
**Gender**					
Male	15 (60%)	16 (64%)	31 (62%)	χ^2^= 0.085	0.771
Female	10 (40%)	9 (36%)	19 (38%)		
**Age (/Years)**					
Mean ± SD.	53.96 ± 14.07	52.16 ± 12.22	53.06 ± 13.07	t = 0.483	0.631
Median (Min. – Max.)	56 (21–73)	52 (25–73)	55 (21–73)		
**Smoking**					
No smoking	16 (64%)	19 (76%)	35 (70%)	χ^2^= 1.318	^MC^p= 0.540
Current smoking	6 (24%)	5 (20%)	11 (22%)		
Previous smoker	3 (12%)	1 (4%)	4 (8%)		
**Weight (kg)**					
Mean ± SD.	79.28 ± 20.33	87.92 ± 25.98	83.60 ± 23.50	U = 243.50	0.180
Median (Min. – Max.)	76 (48–155)	85 (54–174)	79.5 (48 − 174)		
**Height (m)**					
Mean ± SD.	1.68 ± 0.10	1.71 ± 0.11	1.70 ± 0.10	t = 1.294	0.202
Median (Min. – Max.)	1.68 (1.5–1.9)	1.75 (1.5–1.9)	1.7 (1.5–1.92)		
**Body mass index (BMI) (kg/m** ^**2**^ **)**					
Mean ± SD.	28.09 ± 5.74	29.58 ± 6.41	28.83 ± 6.07	U = 267.500	0.382
Median (Min. – Max.)	26.4 (20.8–44.8)	27.7 (19.4–47.2)	27.45(19.4–47.2)		
**Duration on dialysis (/month)**					
Mean ± SD.	45.72 ± 32.37	78.64 ± 55.25	62.18 ± 47.80	U = 196.50*	0.024^*^
Median (Min. – Max.)	48 (3–144)	60 (4–192)	48 (3–192)		
**Vascular access**					
Arteriovenous fistula (AVF)	25 (100%)	24 (96%)	49 (98%)	χ^2^ = 1.020	^FE^p= 1.000
Tunneled hemodialysis catheter	0 (0%)	1 (4%)	1 (2%)		
**Epoetin alfa (EPO)**	15 (60%)	12 (48%)	27 (54%)	χ^2^ = 0.725	0.395
**Folic acid/vitamin B-complex**	5 (20%)	5 (20%)	10 (20%)	χ^2^ = 0.000	1.000
**Type and number of anti-hypertensive drugs**					
Angiotensin Receptor Blockers	8 (32%)	6 (24%)	14 (28%)	χ^2^ = 0.397	0.529
Beta blockers	23 (92%)	25 (100%)	48 (96%)	χ^2^ = 2.083	^FE^*p*=0.490
Centrally acting (alpha methyldopa)	9 (36%)	13 (52%)	22 (44%)	χ^2^ = 1.299	0.254
Alpha Blockers	18 (72%)	15 (60%)	33 (66%)	χ^2^ = 0.802	0.370
Calcium Channel Blockers	23 (92%)	22 (88%)	45 (90%)	χ^2^ = 0.222	^FE^*p*=1.000
**Etiology of end stage renal disease**					
Diabetes mellitus (DM)	4 (16%)	6 (24%)	10 (20%)	χ^2^ = 0.500	0.480
Chronic glomerulonephritis (CGN)	2 (8%)	0 (0%)	2 (4%)	χ^2^ = 2.083	^FE^*p*=0.490
Chronic pyelonephritis	0 (0%)	1 (4%)	1 (2%)	χ^2^ = 1.020	^FE^*p*=1.000
Stones	0 (0%)	1 (4%)	1 (2%)	χ^2^ = 1.020	^FE^*p*=1.000
Unknown	1 (4%)	2 (8%)	3 (6%)	χ^2^ = 0.355	^FE^*p*=1.000
Congenital (Bilateral renal agenesis)	0 (0%)	1 (4%)	1 (2%)	χ^2^ = 1.020	^FE^*p*=1.000
Other (analgesic nephropathy)	4 (16%)	4 (16%)	8 (16%)	χ^2^ = 0.000	^FE^*p*=1.000

SD: Standard deviation t: Student t-test U: Mann Whitney test

χ^2^: Chi square test FE: Fisher Exact MC: Monte Carlo

p: p value for comparing between the two studied groups

*: Statistically significant at *p* ≤ 0.05

### Blood pressure

There were no statistically significant differences in the baseline blood pressure readings between the treatment and control groups. Baseline measurements started from similar levels based on an average of four mid-week pre- and post-dialysis BP readings.

Comparing the systolic blood pressure (SBP) readings between the groups, there was no significant difference in the first month. In the second month, however, there was a meaningful mean decrease in pre-dialysis systolic pressure of (− 14.29 ± 15.32 mmHg) in the treatment group, in comparison with the control group at (-3.28 ± 15.53 mmHg; *p* = 0.017). This trend continued in the third month with a more pronounced decrease in the treatment group (-19.71 ± 12.31 mmHg), compared with the control group (-5.40 ± 14.30 mmHg; *p* < 0.001). A similar pattern occurred in post-dialysis measurements, with the treatment group exhibiting significantly greater reductions in the second and third months than the control group.

Analysis of diastolic blood pressure (DBP) readings shows there are significant trends in both pre- and post-dialysis phases. First, during the first month, no statistically significant evidence of a difference was found between treatment and control groups when considering pre-dialysis DBP reduction (*p* = 0.944). Both exhibited relatively small reductions; the mean reductions were by -3.96 ± 4.60 mmHg (treatment) and by -3.44 ± 7.23 mmHg for the control. From the second month onwards, a clear DBP reduction benefit of the treatment group over the control was obvious, where mean reductions were − 7.63 ± 6.01 mmHg and − 3.48 ± 6.90 mmHg for the treatment and control groups, respectively (*p* = 0.021). This lasted through the third month, with the treatment group still holding a DBP reduction *p* = 0.026, with mean reductions of -9.08 ± 7.83 mmHg versus − 4.32 ± 7.63 mmHg for the treatment and control, respectively. Similarly, the post-dialysis DBP change did not significantly differ between the treatment and control groups in the first month of treatment, with a mean reduction of -2.48 ± 6.73 versus − 0.12 ± 4.80 mmHg for the treatment and control groups, respectively (*p* = 0.178). However; from the second month, there were far greater reductions in the treatment group than in the control group: mean reductions of -6.17 ± 7.39 and − 11.88 ± 9.97 mmHg in the treatment group versus − 0.64 ± 7.08 and − 4.56 ± 8.94 mmHg in the control group in the second and third month respectively (*p* = 0.013 at the second month, *p* = 0.005 at the third month) (Table 2).

**Table 2:** Comparison between the treatment and control groups according to change in blood pressure throughout the study period

		Baseline	1st month	2nd month	3rd month	F (*p*)
**Pre-dialysis SBP (mmHg)**	**Control**	**(** ***n*** ** = 25)**	**(** ***n*** ** = 25)**	**(** ***n*** ** = 25)**	**(** ***n*** ** = 25)**	
	Mean ± SD.	155.5^a^ ± 9.04	154.4^a^ ± 11.10	152.2^a^ ± 13.65	150.1^a^ ± 14.93	F = 1.186 *p* = 0.321
	Median (Min. – Max.)	153 (140–175)	158(120–180)	153(120–175)	150(110–180)	
	**Change**		**-1.08 ± 12.92**	**-3.28 ± 15.53**	**-5.40 ± 14.30**	
	**Treatment**	**(** ***n*** ** = 25)**	**(** ***n*** ** = 25)**	**(** ***n*** ** = 25)**	**(** ***n*** ** = 25)**	
	Mean ± SD.	155.9^a^ ± 14.73	150.3^a^ ± 8.17	142.3^b^ ± 15.57	136.8^b^ ± 11.41	F = 22.304^*^ *p* < 0.001^*^
	Median (Min. – Max.)	150(140–206)	150(130–165)	144(100–170)	139.5(108–165)	
	**Change**		**-5.56 ± 13.46**	**-14.29 ± 15.32**	**-19.71 ± 12.31**	
	**U (p**_**1**_ **)**		252.50 (0.243)	180.50^*^ (0.017^*^)	110.00^*^(< 0.001^*^)	
**Post-dialysis SBP (mmHg)**	**Control**	**(** ***n*** ** = 25)**	**(** ***n*** ** = 25)**	**(** ***n*** ** = 25)**	**(** ***n*** ** = 25)**	
	Mean ± SD.	140.2^a^ ± 9.71	138.6^a^ ± 8.34	137.6^a^ ± 13.27	134.4^a^ ± 14.50	F = 2.670 *p* = 0.077
	Median (Min. – Max.)	140 (130–170)	140 (128–163)	140 (100–168)	135 (110–175)	
	**Change**		**-1.64 ± 4.55**	**-2.64 ± 9.63**	**-5.76 ± 11.84**	
	**Treatment**	**(** ***n*** ** = 25)**	**(** ***n*** ** = 25)**	**(** ***n*** ** = 25)**	**(** ***n*** ** = 25)**	
	Mean ± SD.	144.4^a^ ± 19.04	141.3^a^ ± 14.05	130.0^b^ ± 13.88	121.2^c^ ± 15.29	F = 34.026^*^ *p* < 0.001^*^
	Median (Min. – Max.)	140 (130–207)	140 (110–183)	130 (100–165)	122.5(90–155)	
	**Change**		**-3.12 ± 10.10**	**-14.13 ± 12.51**	**-22.98 ± 16.89**	
	**U (p**_**1**_ **)**		276.00 (0.455)	127.50^*^ (0.001^*^)	116.50^*^ (< 0.001^*^)	
**Pre-dialysis DBP (mmHg)**	**Control**	**(** ***n*** ** = 25)**	**(** ***n*** ** = 25)**	**(** ***n*** ** = 25)**	**(** ***n*** ** = 25)**	
	Mean ± SD.	92.24^a^ ± 5.71	88.80^a^ ± 6.37	88.76^a^ ± 4.23	87.92^a^ ± 8.91	F = 2.772 *p* = 0.064
	Median (Min. – Max.)	90 (80–110)	90 (80–110)	90 (80–98)	90 (65–105)	
	**Change**		**-3.44 ± 7.23**	**-3.48 ± 6.90**	**-4.32 ± 7.63**	
	**Treatment**	**(** ***n*** ** = 25)**	**(** ***n*** ** = 25)**	**(** ***n*** ** = 25)**	**(** ***n*** ** = 25)**	
	Mean ± SD.	91.04^a^ ± 5.25	87.08^b^ ± 5.51	83.46^bc^ ± 5.79	82.0^c^ ± 6.52	F = 19.261^*^ *p* < 0.001^*^
	Median (Min. – Max.)	90 (80–110)	90 (75–98)	83 (68–95)	80 (65–90)	
	**Change**		**-3.96 ± 4.60**	**-7.63 ± 6.01**	**-9.08 ± 7.83**	
	**U (p**_**1**_ **)**		309.00 (0.944)	187.00^*^ (0.021^*^)	190.00^*^ (0.026^*^)	
**Post-dialysis DBP (mmHg)**	**Control**	**(** ***n*** ** = 25)**	**(** ***n*** ** = 25)**	**(** ***n*** ** = 25)**	**(** ***n*** ** = 25)**	
	Mean ± SD.	85.68^a^ ± 6.19	85.56^a^ ± 4.85	85.04^a^ ± 7.23	81.12^b^ ± 8.95	F = 4.104^*^ *p* = 0.017^*^
	Median (Min. – Max.)	85 (80–100)	88 (80–90)	80 (73–105)	80 (68–98)	
	**Change**		**-0.12 ± 4.80**	**-0.64 ± 7.08**	**-4.56 ± 8.94**	
	**Treatment**	**(** ***n*** ** = 25)**	**(** ***n*** ** = 25)**	**(** ***n*** ** = 25)**	**(** ***n*** ** = 25)**	
	Mean ± SD.	87.76^a^ ± 7.55	85.28^a^ ± 4.95	81.50^b^ ± 6.67	75.79^c^ ± 8.90	F = 22.457^*^ *p* < 0.001^*^
	Median (Min. – Max.)	90 (80–110)	88 (78–90)	80 (68–90)	78 (60–90)	
	**Change**		**-2.48 ± 6.73**	**-6.17 ± 7.39**	**-11.88 ± 9.97**	
	**U (p**_**1**_ **)**		249.50 (0.178)	178.00^*^ (0.013^*^)	160.00^*^ (0.005^*^)	

SD: Standard deviation U: Mann Whitney test F: F test (ANOVA) with repeated measures, Sig. bet. periods were done using Post Hoc Test (adjusted Bonferroni)

p: p value for comparing between the different studied periods

p_1_: p value for comparing between Treatment group and Control group in Change

*: Statistically significant at *p* ≤ 0.05

SBP: Systolic Blood Pressure DBP: Diastolic Blood Pressure

Means in the same row with any small common letter ^(a−c)^ are not significant

### Serum homocysteine level

We found a statistically significant reduction in the mean serum level of homocysteine from a baseline of 25.70 ± 9.70 µmol/L to 21.96 ± 9.82 µmol/L at 3 months in the treatment group (*p* = 0.035) with 7 patients (28%) achieving normal serum homocysteine level < 15 µmol/L. In the control arm, however, the mean serum level of homocysteine stayed relatively the same from a baseline level of 24.0 ± 7.17 mmol/l to a three-month level of 26.32 ± 10.39 µmol/L (*p* = 0.236). This is supported by a statistically significant difference (*p* = 0.006) in the changes in homocysteine levels between the treatment and control groups (Table 3).

**Table 3:** Comparison between the treatment and control groups according to the change in laboratory investigations throughout the study period

	Control			Treatment			U (*p*)
	Baseline (*n* = 25)	After 3 months (*n* = 25)	Change	Baseline (*n* = 25)	After 3 months (*n* = 25)	Change	
**Hemoglobin (g/dl)**							
Mean ± SD.	8.93 ± 1.13	9.46 ± 1.46	0.53 ± 1.27	9.55 ± 1.21	9.79 ± 1.22	0.20 ± 0.99	U = 243.5 *p* = 0.258
Median (Min. – Max.)	9.3(6.2–10.5)	9.5(6.7–12.8)	0.4 (-1.6–4)	9.6(6.6–11.6)	10 (6.6–12.2)	0.05 (-2–3.2)	
**t (p**_**0**_**)**	t = 2.091^*^ (p_0_ = 0.047^*^)			t = 0.966 (p_0_ = 0.344)			
**White Blood Cells (10** ^**3**^ **/mm** ^**3**^ **)**							
Mean ± SD.	6.44 ± 2.25	6.23 ± 1.77	-0.21 ± 1.26	6.60 ± 2.85	6.30 ± 1.93	-0.29 ± 1.76	U = 271.50 *p* = 0.568
Median (Min. – Max.)	5.7 (3–11.3)	6.1 (3–9.2)	0 (-3–1.6)	6.6 (2.7–14.7)	6.25(2.8–11.4)	-0.35(-4.3–3.9)	
**t (p**_**0**_**)**	t = 0.824 (p_0_ = 0.418)			t = 0.800 (p_0_ = 0.432)			
**Platelets (10** ^**3**^ **/mm** ^**3**^ **)**							
Mean ± SD.	239 ± 64.11	245.1 ± 67.48	6.04 ± 43.85	211.9 ± 57.36	206.5 ± 54.43	-1.25 ± 55.51	U = 274.0 *p* = 0.603
Median (Min. – Max.)	231(111–346)	253(102–366)	7 (-76–83)	222(132–332)	214.5(96–296)	0.5(-106–116)	
**t (p**_**0**_**)**	t = 0.689 (p_0_ = 0.498)			t = 0.110 (p_0_ = 0.913)			
**Calcium (mg/dl)**							
Mean ± SD.	8.97 ± 0.82	8.83 ± 1.32	-0.14 ± 1.37	9.08 ± 0.84	9.11 ± 0.80	0.04 ± 0.70	U = 295.50 *p* = 0.928
Median (Min. – Max.)	8.9 (7.4–10.2)	9. (4.5–10.8)	-0.1(-5.5–1.7)	9.2 (7.3–10.7)	9.3 (7.5–10.1)	0.25(-1.2–1.3)	
**t (p**_**0**_**)**	t = 0.496 (p_0_ = 0.625)			t = 0.286 (p_0_ = 0.778)			
**Phosphorus (mg/dl)**							
Mean ± SD.	5.88 ± 2.04	5.60 ± 1.46	-0.28 ± 1.76	5.88 ± 1.62	5.83 ± 1.86	-0.21 ± 2.12	U = 265.50 *p* = 0.490
Median (Min. – Max.)	5.6 (3–10.9)	5.2 (3.3–9.5)	0 (-4.8–2.9)	5.9 (1.9–8.4)	5.55(2.5–10.3)	-0.65(-4.1–5.7)	
**t (p**_**0**_**)**	t = 0.808 (p_0_ = 0.427)			t = 0.482 (p_0_ = 0.634)			
**PTH (pg/ml)**							
Mean ± SD.	253.3 ± 168.8	253.6 ± 131.3	0.32 ± 53.70	375.6 ± 350.8	347.3 ± 274.2	-37.17 ± 107.5	U = 238.50 *p* = 0.219
Median (Min. – Max.)	199(95–741)	218(77–630)	6 (-131–113)	293(63–1714)	291(50–1266)	-12 (-448–87)	
**Z (p**_**0**_**)**	Z = 0.215 (p_0_ = 0.830)			Z = 1.400 (p_0_ = 0.161)			
**Homocysteine (µmol/L)**							
Mean ± SD.	24.0 ± 7.17	26.32 ± 10.39	2.32 ± 9.56	25.70 ± 9.70	21.96 ± 9.82	-4.13 ± 9.01	U = 163.00^*^ *p* = 0.006*
Median (Min. – Max.)	23.2(14.5–40.3)	26(10.1–59.4)	0.8(-16.9–36.6)	23.6(14.2–50)	19(8.3–41.2)	-4.7(-22.8–15)	
**t (p**_**0**_**)**	t = 1.215 (p_0_ = 0.236)			t = 2.244^*^ (p_0_ = 0.035^*^)			

SD: Standard deviation U: Mann Whitney test

t: Paired t-test Z: Wilcoxon signed ranks test

p: p value for comparing between Treatment group and Control group in change

p_0_: p value for comparing between Baseline and After 3 months in each group

*: Statistically significant at *p* ≤ 0.05

### Other laboratory results

On the other hand, there were no statistically significant changes observed from baseline and throughout the three months of the study period within each group or between groups as regards serum calcium, phosphorus, or parathyroid hormone (PTH) levels, as shown in Table 3. The CBC characteristics were consistent across all groups, except a modest rise in hemoglobin levels in the control group (8.93 ± 1.13 at baseline vs. 9.46 ± 1.46 after three months; *p* = 0.047). There were no statistically significant differences between the two groups in any CBC markers from baseline to three months.

### Regression analysis for factors affecting serum homocysteine level

A univariate analysis, including several parameters, such as treatment with the study drug, participant demographics, smoking status, anthropometric measurements, months of dialysis, baseline medications, and antihypertensive drugs, was conducted. This univariate analysis concluded that, among these parameters, only the study drug showed a statistically significant association with a reduction in serum homocysteine, with a coefficient of 6.449 and a 95% confidence interval of 1.106–11.792 (*p* = 0.019).

### Regression analysis for factors affecting BP control

Another univariate analysis looking at the effect of the same variables and ultrafiltration volume on the reduction of pre- and post-SBP and DBP readings found the study drug to be also associated with a significant reduction of pre-dialysis SBP with a coefficient B 14.308, 95% CI: 6.626–21.991 (*p* < 0.001).

For post-dialysis SBP, univariate analysis found two possible contributors: methylfolate supplementation and reduction in serum homocysteine. In this regard, there was an association between methylfolate supplementation and post-dialysis SBP reduction with a B coefficient of 17.219 and a 95% CI of 8.865 to 25.573 at *p* < 0.001. Serum homocysteine reduction was suggestively associated with a B coefficient of 0.487 and a 95% CI of 0.002 to 0.972 at *p* = 0.049. Further multivariate regression analysis confirmed methylfolate supplementation as the single statistically significant contributor to post-dialysis SBP reduction with a B coefficient of 15.846 with a 95% CI of 6.967–24.726, p-value 0.001. Serum homocysteine reduction association with post-dialysis SBP did not reach statistical significance in the multivariate analysis, p: 0.356.

It was observed that only supplementation with methylfolate was associated with a reduction in pre-dialysis DBP with a coefficient of 4.763 with a 95% CI of 0.320 to 9.206 (*p* = 0.036), thus suggesting that supplementation of methylfolate had a significant association with reducing pre-dialysis DBP levels. Reduced post-dialysis DBP also exhibited a similar trend of being significantly associated with the administration of methylfolate alone. With a coefficient of 7.315, having a 95% confidence range of (1.877–12.753) (*p* = 0.009), our results would then imply that the supplementation with methylfolate had significantly impacted the post-dialysis DBP reductions of subjects in this study.

While one patient in the treatment group unfortunately experienced an acute myocardial infarction leading to hospitalization and subsequent mortality, the limited overall infrequency of these events prevented statistically significant comparisons between the treatment and control arms.

## Discussion

High prevalence of elevated serum homocysteine concentrations has been observed among patients with ESRD. Therapies that would be effective in reducing homocysteine levels over the long term are less likely to have a cardiovascular benefit in the dialysis population, similar to individuals without kidney disease [26]. Interest in treatment options has recently focused on the use of methylfolate as a potentially superior agent to folic acid in reducing circulating homocysteine concentrations [21]. 

Overall, the most common prescribed classes of antihypertensives in our cohort were the calcium channel blockers in 90% and beta-blockers in 96%, respectively; this was still consistent with data in the 2020 report of the Egyptian renal data system (ERDS) in patients with ESRD [27]. More importantly, the baseline analysis did not show any difference in the classes of antihypertensive medications used by the two study arms.

Comparing baseline and post-intervention BP readings in the treatment group made an important observation. There was a statistically significant reduction in mean serum homocysteine level from 25.70 ± 9.70 mmol/l at baseline to 21.96 ± 9.82 mmol/l after 3 months. On the other hand, there were no significant changes in the control group (p value = 0.236), where the mean values remained almost similar at baseline and after 3 months, with 24.0 ± 7.17 versus 26.32 ± 10.39 mmol/l, respectively.

A statistically significant difference between the levels of homocysteine reduction before and after treatment in the treatment versus the control group was evident (p-value = 0.006), supporting an effective intervention. The median treatment effect for homocysteine was − 4.70 mmol/l, ranging from the lower quartile − 8.90 to -0.70, suggesting a decrease in the homocysteine level post-intervention. Conversely, the control group experienced a modest median increase of merely 0.80 mmol/l (-2.10 to 5.30 IQR), suggesting that there was no substantial change over the observed period.

Our findings generally agree with those of a recent pilot study on the effects of a three-month dietary supplementation of methylfolate on homocysteine plasma levels among diabetic individuals [28]. There are, however, a number of differences noticeable, since previous research was conducted on diabetic subjects with lower baseline homocysteine levels compared to our ESRD cohort. Naturally, this study also met some limitations of a pilot study-like design: it was noncontrolled, and the sample size under study was relatively small.

This magnitude of decrement in serum level suggests that a significant effect from supplementation of methylfolate might be seen in homocysteine levels if continued for 3 months. Our study design was, therefore, more sound in the ESRD patient population and further targeted because our patient groups had higher mean baseline homocysteine levels.

Several studies affirm the previous theory that preference for methylfolate over folic acid is based on the proven efficacy of the former. Methylfolate bypasses the complete folate metabolism route. This pathway has gained significance, assuming that folic acid is poorly bioequivalent to naturally occurring dietary folates, especially in individuals with common folate polymorphisms [29–31]. Multiple reports exist regarding several methylenetetrahydrofolate reductase gene variations, the influence of which could alter the effectiveness of folic acid supplementation [32]. 

To the authors’ best knowledge, this is the first study to investigate the specific role of serum homocysteine reduction among ESRD patients with resistant hypertension. Some studies claimed the involvement of hyperhomocysteinemia as a cause of hypertension [33–35], whereas other studies did not find an association between the lowering effect of homocysteine and the reduction of blood pressure [36]. 

Although the findings here suggest that a reduction in serum homocysteine may possibly, in fact, contribute to improved blood pressure, it is clear that other mechanisms must be involved. Several studies have shown that methylfolate can directly have favorable effects on endothelial function independent of actions on the homocysteine level. An in-vitro study showed a direct effect in that 5-methyltetrahydrofolate directly reduces the production of superoxide by cultured endothelial cells, an outcome that has antioxidant properties [37]. Another study indicated that methylfolate supplementation prevented an increase in endothelial superoxide production induced by increased homocysteine levels [38]. 

Limitations of the current trial include the lack of blinding and the absence of a placebo, in addition to the modest sample size. Given the consistent rise in the treatment effect over time, a cumulation advantage is plausible. This would highlight the need for future large randomized studies, a placebo-controlled, double-blind design, or a crossover design to investigate the long-term effectiveness, optimal dose, and optimal duration of methylfolate for the long-term successful treatment of elevated blood pressure in this patient group and to increase internal validity and within-subject power.

## Conclusions

Our study, which used three months of daily treatment with methylfolate 800 mcg and methylcobalamin 1000 mcg in ESRD patients with resistant hypertension, yielded favorable outcomes. During the three-month trial period, the treatment group revealed a statistically significant reduction in serum homocysteine as well as pre- and post-dialysis blood pressure readings. The results are intriguing; nevertheless, more investigations in large trial settings will be required to validate our findings in these patients’ population.

## Data Availability

The datasets used and analyzed during the current study are available from the corresponding author on reasonable request.

## Abbreviations

BP: Blood pressure
BMI: Body mass index
CVDs: Cardiovascular diseases
CGN: Chronic glomerulonephritis
CBC: Complete blood count
DM: Diabetes mellitus
DBP: Diastolic blood pressure
ERDS: Egyptian renal data system
ESRD: End-stage renal disease
EPO: Epoetin Alpha
ESA: Erythropoietin stimulating agent
H-type hypertension: Hyperhomocysteinemia associated hypertension
PTH: Parathyroid hormone
SBP: Systolic blood pressure
